# Dual effectiveness of *Alternaria* but not *Fusarium* mycotoxins against human topoisomerase II and bacterial gyrase

**DOI:** 10.1007/s00204-016-1855-z

**Published:** 2016-09-28

**Authors:** Katharina Jarolim, Giorgia Del Favero, Doris Ellmer, Timo D. Stark, Thomas Hofmann, Michael Sulyok, Hans-Ulrich Humpf, Doris Marko

**Affiliations:** 1 Department of Food Chemistry and Toxicology, Faculty of Chemistry, University of Vienna, 1090 Vienna, Austria; 2 Chair of Food Chemistry and Molecular Sensory Science, Technical University of Munich, 85354 Freising, Germany; 3 Department IFA-Tulln, University of Natural Resources and Life Sciences Vienna (BOKU), 3430 Tulln, Austria; 4 Institute of Food Chemistry, Westfälische Wilhelms-Universität Münster, 48149 Münster, Germany

**Keywords:** Topoisomerase II, Gyrase, Mycotoxins, *Alternaria*, *Fusarium*

## Abstract

Type II DNA-topoisomerases (topo II) play a crucial role in the maintenance of DNA topology. Previously, fungi of the *Alternaria* genus were found to produce mycotoxins that target human topo II. These results implied the question why a fungus should produce secondary metabolites that target a human enzyme. In the current work, the homology between human topo II and its bacterial equivalent, gyrase, served as basis to study a potential dual inhibition of both enzymes by mycotoxins. A total of 15 secondary metabolites produced by fungi of the genera *Alternaria* and *Fusarium* were assessed for their impact on topo II of human and bacterial origin in the decatenation and the supercoiling assay, respectively. In line with the theory of dual topo II inhibition, six of the tested *Alternaria* mycotoxins were active against both enzymes, the dibenzo-α-pyrones alternariol (AOH) and alternariol monomethyl ether (AME), as well as the perylene-quinones altertoxin I (ATX I) and II (ATX II), alterperylenol (ALP) and stemphyltoxin III (STTX III). The *Alternaria* metabolites altersetin (ALN), macrosporin (MAC), altenusine (ALS) and pyrenophorol (PYR) impaired the function of human topo II, but did not show any effect on gyrase. The potency to inhibit topo II activity declined in the row STTX III (initial inhibitory concentration 10 µM) > AOH (25 µM) = AME (25 µM) = ALS (25 µM) = ATX II (25 µM) > ALN (50 µM) = ATX I (50 µM) > ALP (75 µM) = PYR (75 µM) > MAC (150 µM). Inhibition of gyrase activity was most pronounced for AOH and AME (initial inhibitory concentration 10 µM) followed by ATX II (25 µM) > ATX I = ALP = STTX III (50 µM). In contrast, none of the investigated *Fusarium* mycotoxins deoxynivalenol (DON), fumonisin B1, fusarin C and moniliformin, as well as the *Alternaria* metabolite tentoxin, had any impact on the activity of neither human nor bacterial topo II.

**Electronic supplementary material:**

The online version of this article (doi:10.1007/s00204-016-1855-z) contains supplementary material, which is available to authorized users.

## Introduction

DNA-topoisomerases are essential enzymes that play a fundamental role in the maintenance of cellular DNA topology, i.e., the integrity of DNA during replication or chromosomal segregation (Champoux and Dulbecco 1972; DiNardo et al. 1984). According to their respective catalytic mechanism of action, they are divided into type I and type II topoisomerases (Liu et al. 1980). The dimeric topoisomerase (topo) II acts as decatenase, introducing transient double-strand breaks into the DNA substrate, allowing the passage of an intact DNA double-strand and resealing the break afterward (Hsieh and Brutlag 1980; Liu et al. 1980). While a wanted effect in cancer therapy, the functional disturbance of human topo II by food constituents may imply severe adverse effects (McClendon and Osheroff 2007).

**Fig. 1:**
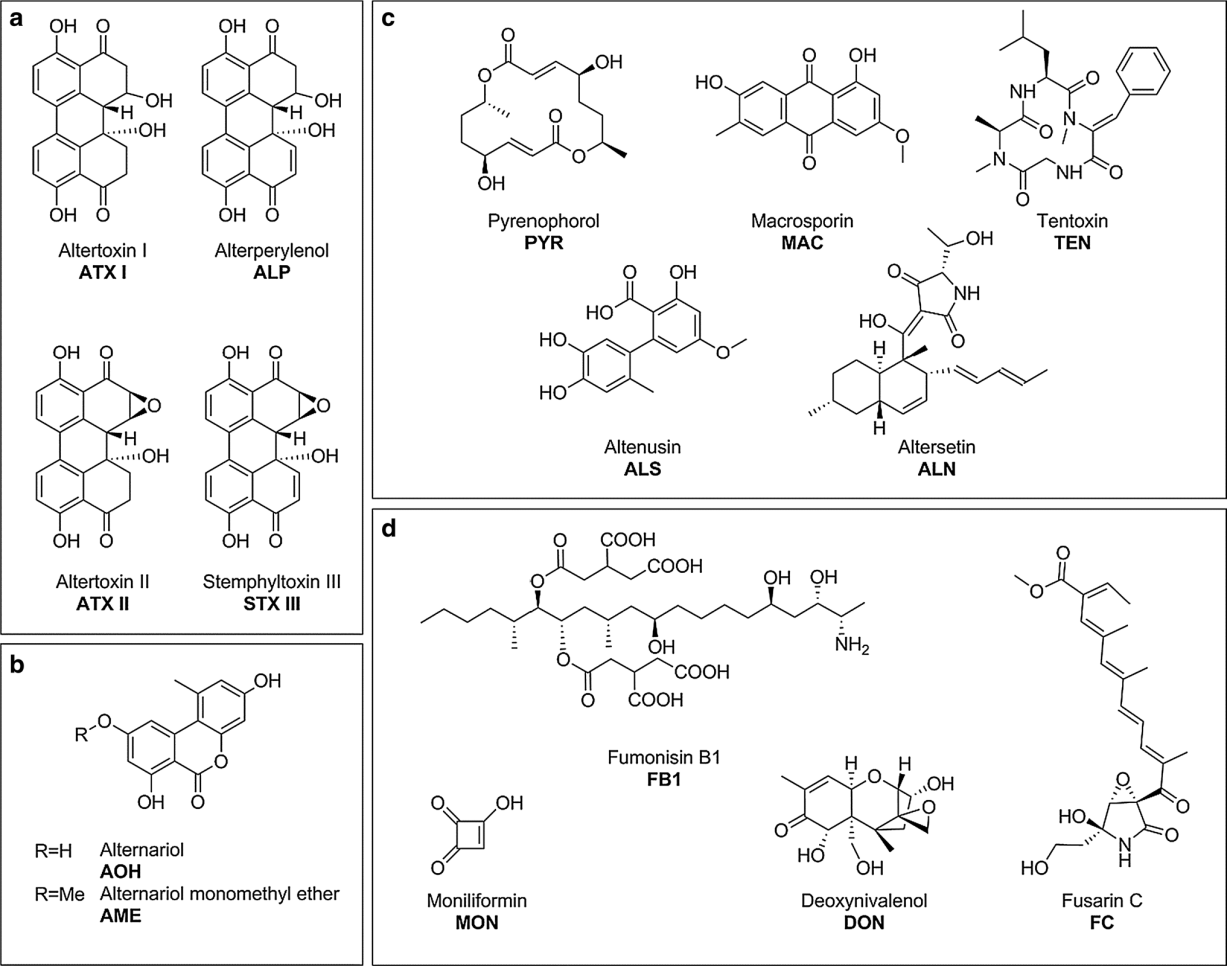
Chemical structures of secondary metabolites produced by *Alternaria* and *Fusarium* fungi. Molecules are grouped according to structure and producing organisms in **a** perylene-quinone-type, **b** dibenzo-α-pyrones both from *Alternaria* and **c** other *Alternaria* mycotoxins, **d** four secondary metabolites produced by *Fusarium* spp

In previous studies, three secondary metabolites produced by food-contaminating fungi of the genus *Alternaria,* the dibenzo-α-pyrones AOH and AME, as well as the perylene-quinone ATX II, were found to inhibit the activity of human topo II in vitro (Fehr et al. 2009; Tiessen et al. 2013). Based on these results, the question arose what might prompt a mold to produce secondary metabolites that target human enzymes. Since a fungus faces a variety of microorganisms within its habitat, against which it must compete, the formation of toxic secondary metabolites that are detrimental to potential predators is discussed as defense method to ensure proper growth and propagation (Magan and Aldred 2007). The production of antibiotics is a mechanism of antibacterial defense that has been observed for many genera of fungi imperfecti, the most prominent being *Aspergillus* and *Penicillium* spp. (Lancini et al. 1995). A common target for antibiotic pharmaceuticals is gyrase, a bacterial topo II (Alt et al. 2011; Gellert et al. 1976b). Since gyrase shares a high degree of homology with human topo II, the previously observed interaction between *Alternaria* mycotoxins and human topo II was taken as indication of a potential dual inhibition. To consider this question, 15 secondary metabolites produced by *Alternaria* and *Fusarium* fungi were investigated for their effect on the activity of topo II of both human and bacterial origin. These molds grow as endophytes on host plants worldwide, contaminating a variety of food and feed (Kharwar et al. 2014; Placinta et al. 1999). The two fungi genera synthesize chemically very diverse secondary metabolites, as can be seen with an exemplarily selection in Fig. 1. Some of the tested *Alternaria* mycotoxins share structural features, for instance the perylene-quinone-type toxins ATX I, ATX II, ALP and STTX III (Fig. 1a), which differ by an epoxy group and/or a double bond. The dibenzo-α-pyrones AOH and AME (Fig. 1b), also produced by *Alternaria* fungi, distinguish themselves only by a methyl group. The further *Alternaria* metabolites that were assessed in this study constitute a chemically heterogeneous group, composed of the macrodiolide PYR, the anthraquinone MAC, the tetrapeptide TEN, the tetramic acid derivative ALN and the biphenyl ALS (Fig. 1c). The investigated *Fusarium* metabolites include the trichothecene DON, the polyketide FB1, which is characterized by an eicosane backbone, the sesquiterpenoid FC and the semiquadric acid MON (Fig. 1d). In the present work, these 15 secondary fungal metabolites were investigated for their potential interference with type II topoisomerases of human and bacterial origin in cell-free experimental setups. Since most of the selected perylene-quinones are not commercially available, these compounds were isolated from rice inoculated with *Alternaria alternata* cultures.

## Experimental procedures

### Cell culture and fungus cultivation

The human colon adenocarcinoma cell line HT29 and the *Alternaria alternata* strain DSM 62010 were obtained from the German Collection of Microorganisms and Cell Cultures GmbH (DSMZ, Braunschweig, Germany). Cells were cultured in Dulbecco’s modified Eagle medium (DMEM; GIBCO Invitrogen, Karlsruhe, Germany) supplemented with 10 % FBS and 1 % P/S and maintained in incubators at 37 °C and 5 % CO_2_. Cultivation of the *Alternaria* strains was performed on moist, autoclaved rice, as described previously (Schwarz et al. 2012b).

### Isolation and identification of *Alternaria* perylene-quinone-type mycotoxins from inoculated rice cultures

Due to lack of commercially available ATX I, ATX II, ALP and STTX III, the toxins were isolated from *Alternaria*-infested rice. Ethyl acetate extracts were prepared after 21 days of incubation from rice which was inoculated with *Alternaria alternata* strain DSM 62010 as described in Schwarz et al. (2012a). Fractionation of the ethyl acetate extracts by solid-phase extraction (SPE) and isolation of the toxins with HPLC were conducted according to Jarolim et al. (2016). For isolation individual fractions were collected, concentrated under reduced pressure (40 °C), and freeze-dried in duplicate, yielding ATX II and STTX III in high purities (>97 % HPLC–UV, 270 nm). ATX I and ALP were re-purified using the HPLC system and column mentioned in Jarolim et al. (2016) and the following gradient. Monitoring the effluent (4.2 ml/min) at 350 nm, chromatography was performed starting with a mixture (80/20, v/v) of aqueous formic acid (0.1 % in H_2_O) and MeCN, the MeCN content was increased to 45 % within 16 min and to 70 % within 4 min, followed by column washing and re-equilibration. Individual fractions were collected, concentrated under reduced pressure (40 °C), and freeze-dried in duplicate, yielding ATX I and ALP (>97 % HPLC–UV, 270 nm).

Aliquots of the isolated fractions were analyzed by means of UPLC–TOF–MS^e^ on a Waters Synapt G2-S HDMS mass spectrometer (Waters, Manchester, UK) coupled to an Acquity UPLC core system (Waters, Milford, MA, USA) equipped with a 2 × 150 mm, 1.7 µm, BEH C18 column (Waters, Manchester, UK) consisting of a binary solvent manager, sample manager and column oven. Operated with a flow rate of 0.4 ml/min at 50 °C, the following gradient was used for chromatography: Starting with a mixture (10/90, v/v) of aqueous formic acid (0.1 % in H_2_O) and MeCN (0.1 % formic acid), the MeCN content was increased to 100 % within 4 min and, then, kept constant for 0.5 min. Scan time for the MS^e^ method (centroid) was set to 0.1 s. Analyses were performed in the negative ESI and the high-resolution mode using the following ion source parameters: capillary voltage −2.5 kV, sampling cone 30 V, source offset 20 V, source temperature 150 °C, desolvation temperature 450 °C, cone gas 30 l/h, nebuliser 6.5 bar and desolvation gas 850 l/h. Data processing was performed by using MassLynx 4.1 SCN 9.16 (Waters, Manchester, UK) and the elemental composition tool for determining the accurate mass. All data were lock mass corrected on the pentapeptide leucine enkephaline (Tyr-Gly-Gly-Phe-Leu, *m/z* 554.2615, [M–H]^−^) in a solution (1 ng/µl) of MeCN/0.1 % formic acid (1/1, v/v). Scan time for the lock mass was set to 0.3 s, an interval of 15 s and 3 scans to average with a mass window of ±0.3 Da. Calibration of the Synapt G2-S in the range from *m/z* 50 to 1200 was performed using a solution of sodium formiate (5 mmol/l) in 2-propanol/H_2_O (9/1, v/v). The UPLC and Synapt G2-S systems were operated with MassLynx™ software (Waters).

All 1D and 2D NMR measurements (^1^H, ^1^H-^1^H-gCOSY, gHSQC, gHMBC and ^13^C) were performed on an Avance III 500 MHz spectrometer with a CTCI probe (Bruker, Rheinstetten, Germany).

ATX I was identified by UPLC-ESI-TOF-MS and NMR as previously described in Jarolim et al. (2016). The structure of the isolated ATX II was described in detail by Schwarz et al. (2012b). ATX II was identified with an *m/z* of 349.0714 ([M–H]^−^, calculated for C_20_H_13_O_6_, 349.0712, +0.6 ppm) in the (–) HRESIMS. Data of the ^1^H NMR and ^13^C NMR of ALP and STTX III are summarized in the supplementary information (Tables S2–S5) and were in line to Hradil et al. (1989); and Stack and Prival (1986). ALP was identified with an *m/z* of 349.0714 ([M-H]^−^, calculated for C_20_H_13_O_6_, 349.0712, +0.6 ppm) in the (–) HRESIMS *m/z*. ^1^H NMR and ^13^C NMR data are summarized in appendix and in line to Hradil et al. (1989); and Okuno et al. (1983). STTX III was identified with an *m/z* of 347.0557 ([M–H]^−^, calculated for C_20_H_11_O_6_, 347.0556, +0.3 ppm) in the (–) HRESIMS. ^1^H NMR and ^13^C NMR data of ALP and STTX III are summarized in the electronic supplementary information (Tables S1–S5) and were in line to Stack and Mazzola (1989).

### Secondary metabolites from *Alternaria* and *Fusarium* spp.

The *Alternaria* dibenzo-α-pyrones AOH and AME and the *Fusarium* trichothecene DON were bought from Sigma–Aldrich (MI, USA). Macrosporin, tentoxin, altenusin and pyrenophorol were obtained from Enzo Life Sciences (Lausen, Switzerland). Altersetin was purchased from AnalytiCon Discovery GmbH (Potsdam, Germany). FB_1_ and FC were isolated from *Fusarium verticillioides* MRC 826 and *Fusarium verticillioides* MRC 0712 as described in the literature (Hübner et al. 2012; Kleigrewe et al. 2011). MON was synthesized according to Lohrey et al. (2011).

### Decatenation assay

The interference of the tested secondary fungal metabolites with human topoisomerase II α was determined using the decatenation assay. Briefly, this assay is based on the measurement of the ability of topo II α enzyme to decatenate a fragment of catenated kDNA. The topo II poison and anticancer drug etoposide was used as positive control (Ross et al. 1984). A gel with 1 % agarose (w/v) was prepared using tris–acetate-EDTA (TAE) puffer containing 20 mM tris, 1 mM EDTA and 9.6 mM acetic acid. The reaction mixture with a final volume of 20 µl contained 0.5 U human topoisomerase II α, 200 ng kDNA, the test substance with a solvent concentration of 5 % EtOH (in case of perylene-quinones due to better stability) or DMSO and complete Topo II Assay Buffer from TopoGEN. The solution was allowed to react for 1 h at 37 °C, and the reaction was stopped on ice by adding loading buffer (25 % glycerol, 0.125 % bromphenolblue) with 5 % N-lauroylsarkosin. For dyeing of DNA bands, the gel was incubated for 20 min with 10 mg/l ethidium bromide solution. For quantification purposes, the fluorescence intensities of the decatenated kDNA bands at the lower end of the gels were used. The fluorescence signal was detected with the ImageQuant LAS-4000 (GE Healthcare Life Sciences, Buckinghamshire, England). Catenated DNA was quantified using Fujifilm Image Gauge software (Fujifilm Medical Systems USA, Inc., Connecticut, USA). For quantification the fluorescence intensities were normalized to solvent control (=100 %).

### Gyrase supercoiling assay

The secondary metabolites were tested for their potential impact on the activity of the bacterial topo II gyrase with the gyrase supercoiling assay, where the difference in mobility between relaxed and supercoiled closed circular pUC19 was used (Gellert et al. 1976a). Gyrase from *E. coli* and relaxed 2686-bp-long puUC19 were purchased from New England Biolabs (Frankfurt am Main, Germany). A gel with 1 % agarose (w/v) was prepared using tris-acetat-EDTA (TAE) puffer containing 20 mM Tris, 1 mM EDTA and 9.6 mM acetic acid. Reaction mixture for determination of gyrase inhibition with a final volume of 20 µl contained 0.5 U gyrase, 250 ng pUC19, 35 mM Tris HCl (pH 7.5), 24 mM KCl, 4 mM MgCl_2_, 2 mM DTT, 1.75 mM ATP, 5 mM spermidine, 0.1 mg/ml BSA and 6.5 % glycerol. The solution was allowed to react for 60 min at 37 °C, and the reaction was stopped on ice by adding loading buffer (25 % glycerol, 0.125 % bromphenolblue) with 5 % *N*-lauroylsarkosin. For dyeing of DNA bands, the gel was incubated for 20 min with 10 mg/l ethidium bromide solution. The fluorescence signal was detected with the ImageQuant LAS-4000 (GE Healthcare Life Sciences, Buckinghamshire, England). Catenated DNA was quantified using Fujifilm Image Gauge software (Fujifilm Medical Systems USA, Inc., Connecticut, USA). Activity of gyrase was assessed according to mobility shifts in the DNA pattern on the gel. Relaxed pUC19 demonstrated a lower migrating distance in the gel compared to the supercoiled analogue which was generated in case of active gyrase. The aminocoumarin antibiotic novobiocin (50 µM) was used as positive control (Gellert et al. 1976b). Quantification of the DNA bands via fluorescence intensity, as performed in the decatenation assay, was not applicable for this method because of the high degree of band multiplicity, presumably due to topoisomeres. Hence, the quantification was done by evaluating the observed initial inhibitory concentrations on the gels of at least three independent experiments and an average initial inhibitory concentration was calculated.

## Results

### Assessment of the impact of selected secondary metabolites from *Alternaria* and *Fusarium* spp. on the activity of human topo II α

**Fig. 2:**
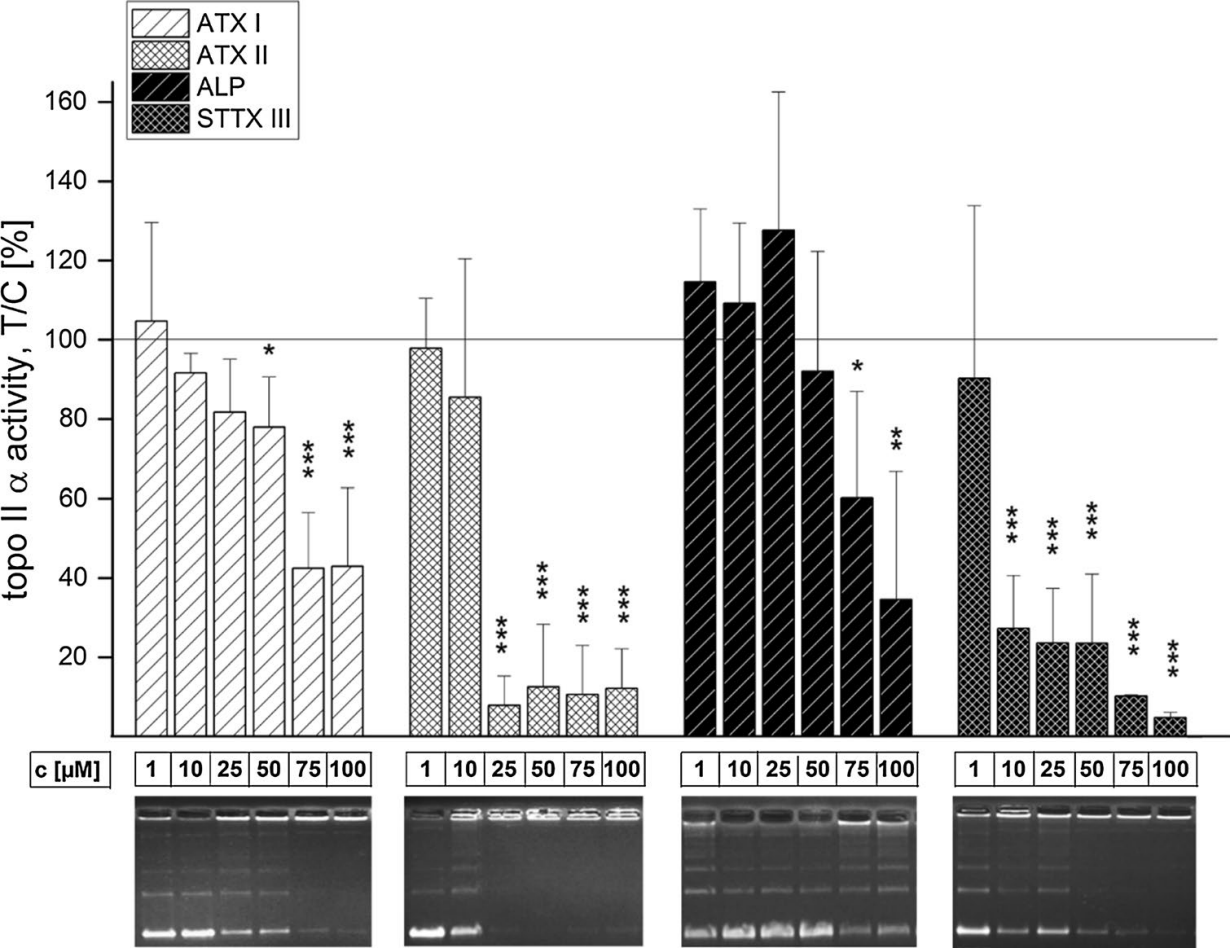
Impact of *Alternaria* mycotoxins ATX I, ATX II, ALP and STTX III on the activity of human topo II α in the decatenation assay. Depicted are the fluorescence intensities of the catenated kDNA, normalized to solvent control (5 % EtOH) and representative gels. Bars refer to mean values ± standard deviation of at least three independent experiments. Significance levels refer to comparison with the respective smallest concentration of each substance ***p < 0.001, **p < 0.01, *p < 0.5)

**Fig. 3:**
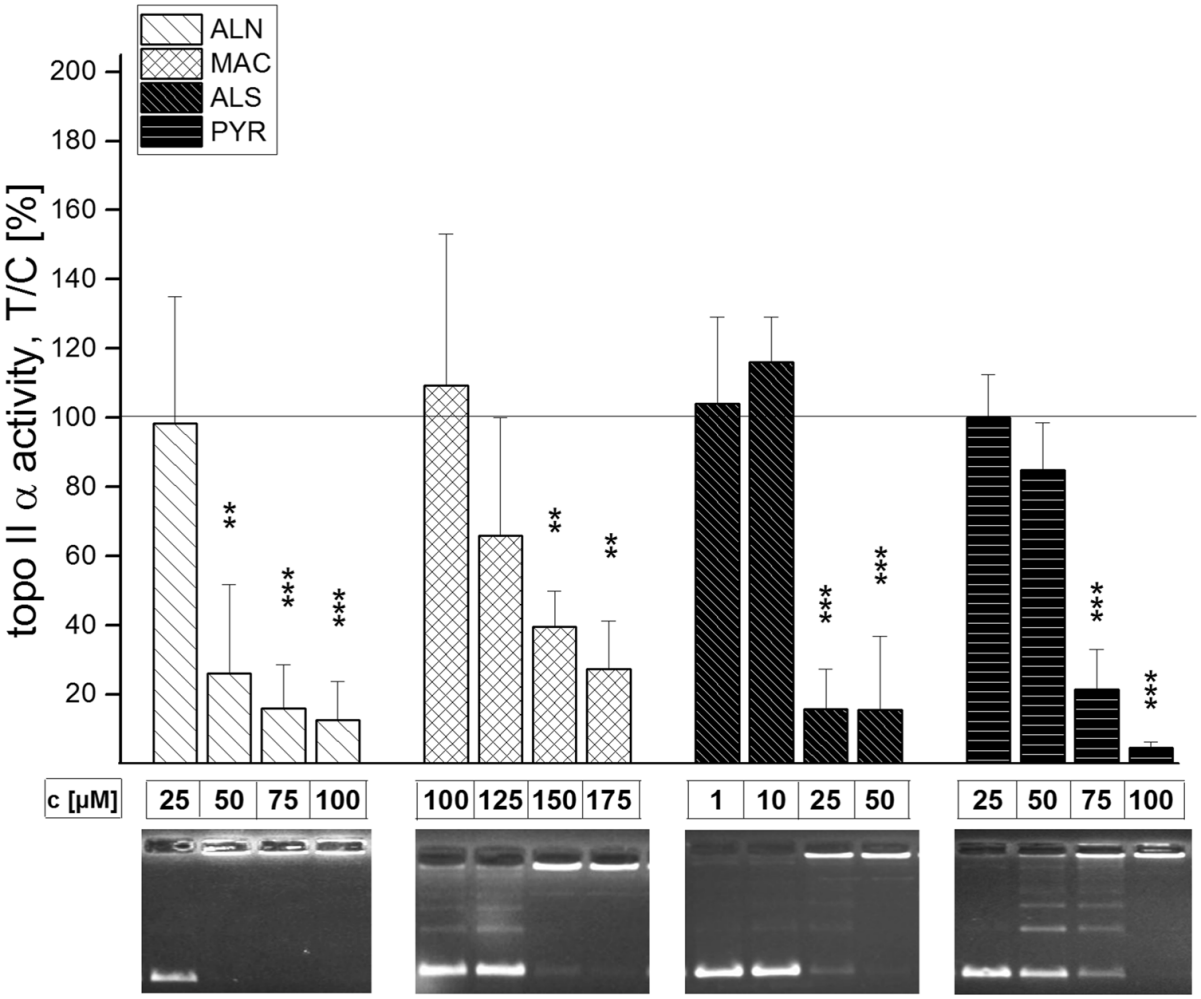
Impact of *Alternaria* mycotoxins ALN, MAC, ALS and PYR on the activity of human topo II α in the decatenation assay. Depicted are the fluorescence intensities of the catenated kDNA, normalized to solvent control (5 % EtOH) and representative gels. Bars refer to mean values ± standard deviations of at least three independent experiments. Significance levels refer to comparison with the respective smallest concentration of each substance ***p < 0.001, **p < 0.01, *p < 0.5)

**Fig. 4:**
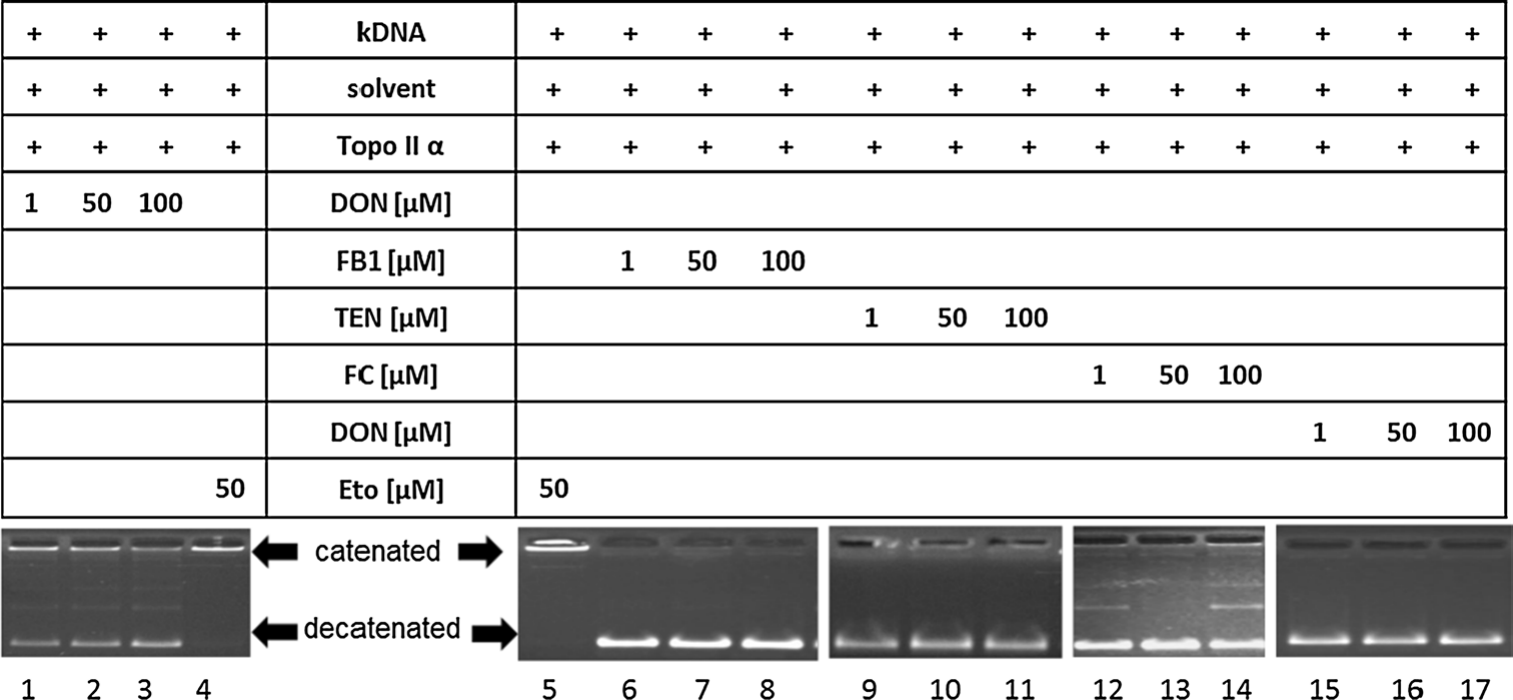
Impact of secondary metabolites of *Fusarium* spp. on the activity of human topo II α in the decatenation assay. Depicted are representative gels of DON (*lanes 1*–*3*), FB1 (*lanes 6*–*8*), TEN (lanes 9–11), FC (*lanes 12*–*14*) and MON (*lanes 15*–*17*) with concentrations of 1, 50 and 100 µM each. Etoposide (50 µM) served as positive control (*lanes 4, 5*)

The effect of 15 secondary metabolites of *Alternaria* and *Fusarium* fungi on the activity of human topo II α was evaluated with the decatenation assay (Table 1). Incubation of human topo II α and kDNA for 1 h in the presence of all four *Alternaria* perylene-quinone-type mycotoxins decreased the activity of the enzyme in a concentration-dependent manner (Fig. 2). ATX I led to a significant decrease at 50 µM and was thus a weaker inhibitor than ATX II, for which an initial inhibitory concentration of 25 µM was observed. ALP, which showed the weakest impact on topo II α of the four compounds, significantly decreased topo II α activity at 75 µM. The most potent inhibitor in the decatenation assay was STTX III with an initial inhibitory concentration of 10 µM. Of the further five *Alternaria* metabolites tested in this assay, ALN, MAC, ALS, PYR (Fig. 3) and TEN (Fig. 4), the first four also showed inhibitory effects on human topo II α. Among these, ALS had the highest potency to suppress topo II-mediated decatenation with an initial inhibitory concentration of 25 µM, thus comparable to ATX II. According to the initial inhibitory concentrations in the decatenation assay, the potency for topo II inhibition declined in the row STTX III (10 µM) > ALS (25 µM) = ATX II (25 µM) > ALN (50 µM) = ATX I (50 µM) > ALP (75 µM) = PYR (75 µM) > MAC (150 µM). Of the four *Fusarium* metabolites that were examined, FB1, MON, DON and FC, none was tested positive for inhibition of the decatenating activity of topo II α up to a concentration of 150 µM (Fig. 4).

**Table 1:** Effect of *Alternaria* and *Fusarium* toxins of the activity of type IIA topoisomerases human topo II α and bacterial gyrase

	Initial inhibitory conc. (μM)	
	h topo II α	Gyrase
*Alternaria* spp.		
Perylene chinones		
ATX I	++	+++
ATX II	++++	++++
ALP	++	+++
STTX III	+++++	+++
Dibenzo-α-pyrones		
AOH	++++^a^	+++++
AME	++++^a^	+++++
Others		
ALN	+++	–
ALS	++++	–
PYR	+++	–
MAC	–	–
TEN	–	–
*Fusarium* spp.		
FB1	–	–
FC	–	–
MON	–	–
DON	–	–

Effects of mycotoxins on human recombinant topo II α (h topo II α) and bacterial gyrase based on at least three independent experiments (initial inhibition at: 10 µM (+++++), 25 µM (++++), 50 µM (+++), 75 µM (++) and 150 µM (+); no inhibition up to 150 µM (–))

^a^Fehr et al. (2009)

### Assessment of the impact of selected secondary metabolites from *Alternaria* and *Fusarium* spp. on the activity of bacterial gyrase

**Fig. 5:**
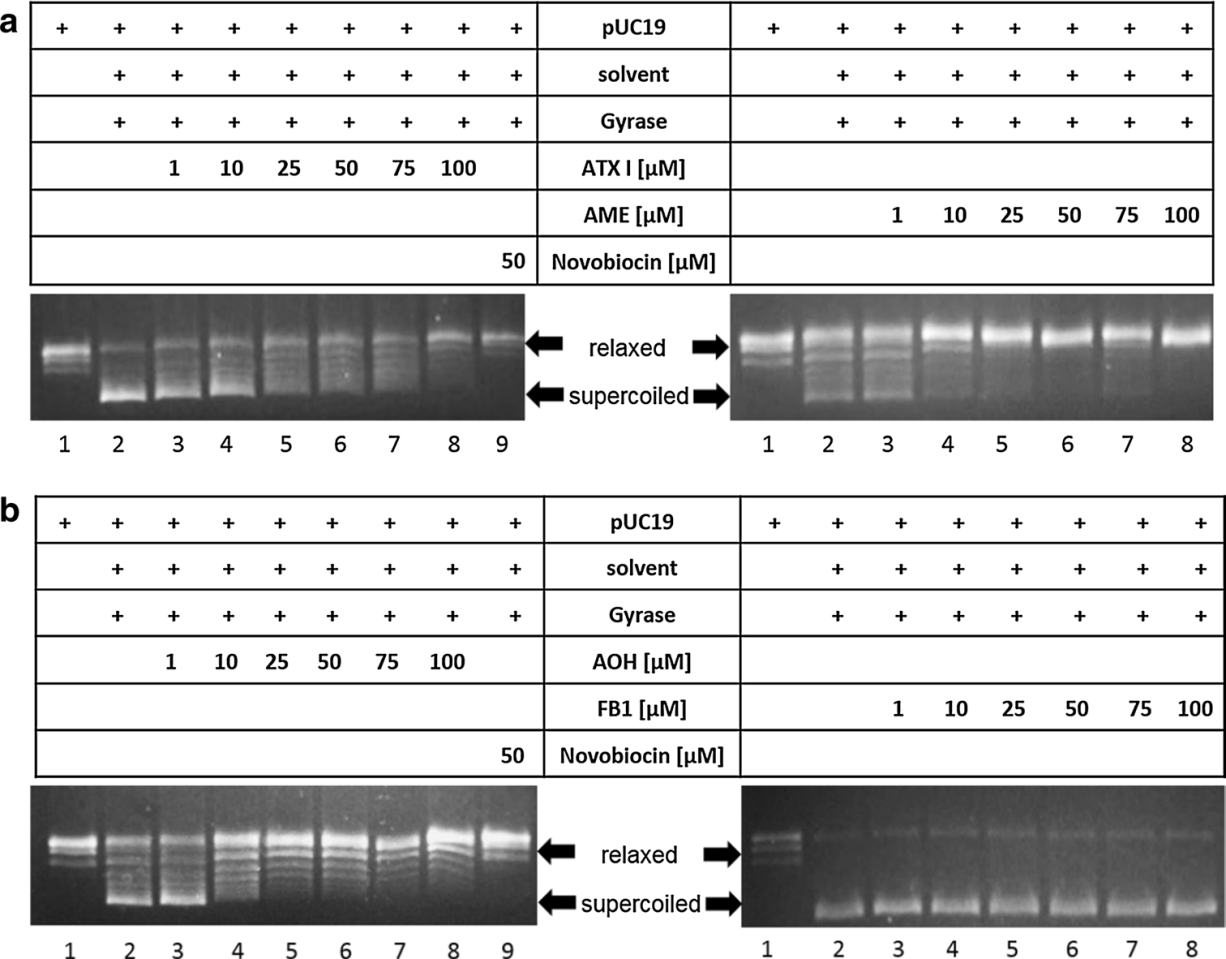
Impact of secondary metabolites produced by *Alternaria* and *Fusarium* fungi on bacterial gyrase. Effect of **a** *Alternaria* perylene-quinone-type mycotoxin ATX I and dibenzo-α-pyrone AME and of **b** *Alternaria* mycotoxin AOH and *Fusarium* mycotoxin FB1 on the supercoiling activity of bacterial gyrase. *Lane 1* relaxed pUC19 without gyrase, *lane 2* addition of 0.5 U gyrase, *lanes 3*–*8* gyrase and toxins in the concentration range 1–100 µM in solvent (5 %), *lane 9* gyrase and novobiocin as positive control

**Fig. 6:**
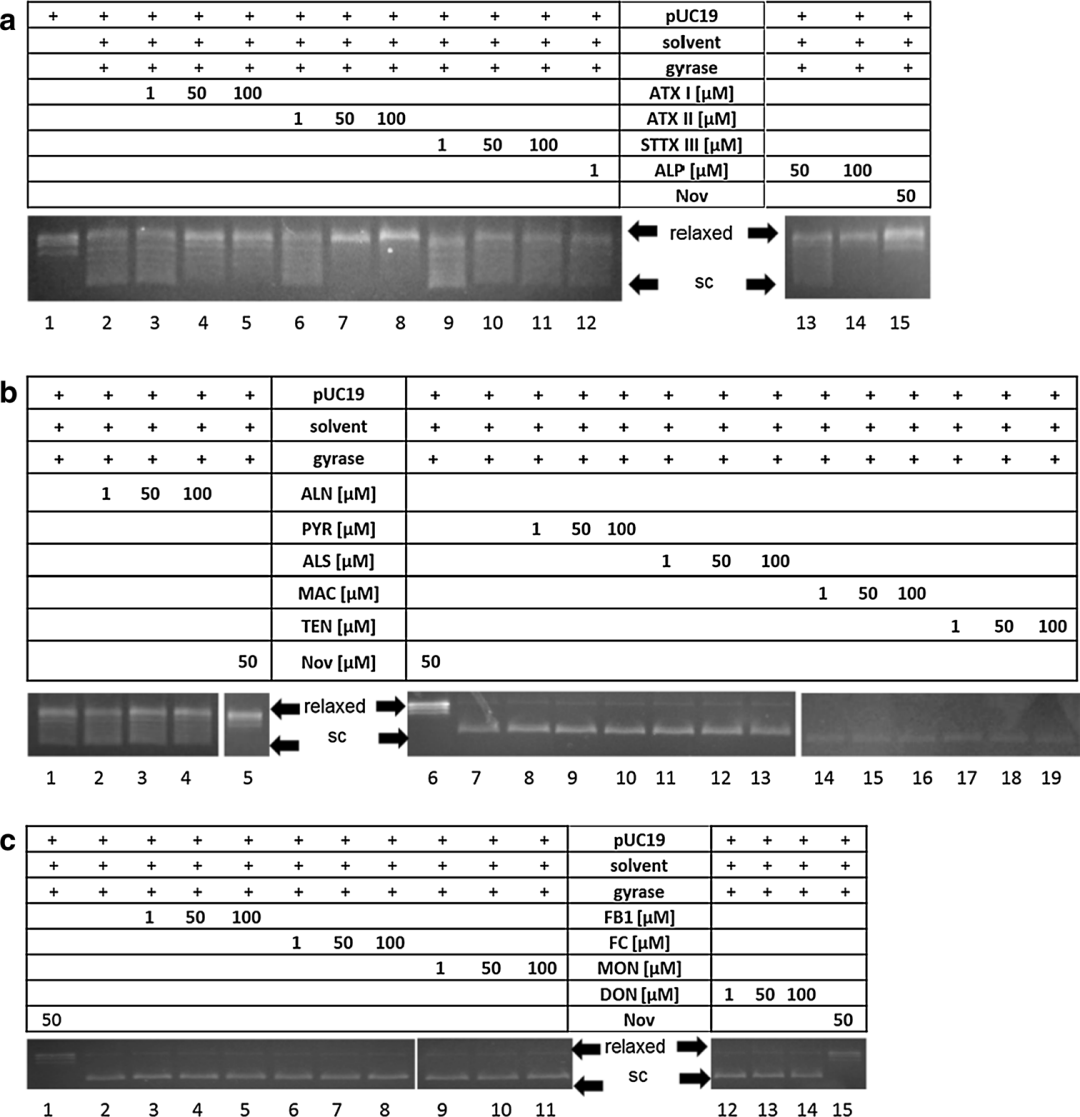
Impact of secondary metabolites produced by *Alternaria* and *Fusarium* fungi on bacterial gyrase. **a** The perylene-quinone derivatives ATX I (*lanes 3*–*5*), ATX II (*lanes 6*–*8*), ALP (*lanes 9*–*11*) and STTX III (*lanes 12*–*14*), **b** *Alternaria* metabolites ALN (*lanes 2*–*4*), PYR (*lanes 8*–*10*), ALS (*lanes 11*–*13*), MAC *(lanes 14*–*16)* and TEN (*lanes 17*–*19*). In **c** gels of FB1 (*lanes 3*–*5*), FC (*lanes 6*–*8*), MON (*lanes 9*–*11*) and DON (*lanes 12*–*14*) are depicted. Novobiocin (50 µM) was used as positive control

In order to evaluate if the effects on human topo II were reflected with respect to interference with the bacterial homologue, the impact of the substances on gyrase from *E. coli* was measured with the gyrase supercoiling assay. This gel-based assay allows an estimation of a substance’s inhibitory potential by assessing the migration of the plasmid pUC19, which is converted from the relaxed topoisomere to the more condensed and thus faster migrating supercoiled form by active gyrase. In Fig. 5 four exemplary gels are depicted of the three active *Alternaria* metabolites ATX I, AOH and AME, which reduced the supercoiling activity of gyrase to different extents, as well as the *Fusarium* mycotoin FB1, which did not show any targeting of gyrase. All four perylene-quinones, ATX I, ATX II, ALP and STTX III, impaired the activity of the enzyme (Fig. 6a). None of the other tested compounds, neither *Alternaria* nor *Fusarium* metabolites, had any effect on the activity of gyrase (Fig. 6b, c). Lane 1 of each gel represents the relaxed DNA plasmid, pUC19, without addition of enzyme or toxin, in lane 2 the respective solvent (DMSO) and gyrase were added. Lanes 3–8 and 3–19 show the addition of the tested compounds in a concentration range from 1 µM to 100 µM. Novobiocin (50 µM) was used as positive control (Figs. 5, lane 9, 6, lane 15). The conversion from the initially relaxed DNA plasmid to the supercoiled form catalyzed by gyrase can be seen when comparing lanes 1 (without gyrase) and 2 (with gyrase). With increasing concentrations of each of the active toxins, ATX I, ATX II, ALP, STTX III, AOH or AME, a shift from the supercoiled plasmid to the relaxed form was observable, indicative for an inhibition of the supercoiling activity of gyrase. The most pronounced inhibitory effects on gyrase activity were observed for the dibenzo-α-pyrones, AOH and AME (initial inhibitory concentrations of 10 µM), followed by the altertoxin ATX II (25 µM) and ATX I, ALP and STTX III (50 µM; Table 1).

## Discussion

Topo II plays an important role in the maintenance and alteration of the topological structure of DNA in the course of cellular processes like replication or chromosomal segregation. Due to its involvement in this broad range of vital biological processes, compounds that interfere with the proper function of the enzyme pose a potential health threat. Previously three mycotoxins produced by the endophytic mold of the genus *Alternaria*, the perylene-quinone derivative ATX II and the dibenzo-α-pyrones AOH and AME, have been described to affect the function of human topo II (Fehr et al. 2009; Tiessen et al. 2013). These results opened a wider research field focused on the effect of fungal secondary metabolites on this important enzyme family. In the present work, 15 secondary metabolites, 11 of which are produced by *Alternaria* spp. and 4 by *Fusarium* spp., were investigated for their effects on human topo II α (Fig. 1). Except for TEN, all the secondary *Alternaria* metabolites inhibited the activity of human topo II α in the decatenation assay in a concentration range between 1 and 150 µM (Figs. 2, 3, 4). Within these ten topo II-targeting metabolites, no correlation between structural characteristics and inhibition potential was observable, except for the *Alternaria* perylene-quinone derivatives. Of the four assessed compounds that belong to this class, ATX II and STTX III revealed the most pronounced impact on the activity of human topo II α. Both mycotoxins contain an epoxide group, whereas their structural analogues ATX I and ALP, weaker inhibitors, lack this moiety (Fig. 1a). The correlation between the presence of an epoxide and, as a consequence thereof, an enhancement of the respective inhibitory potential was restricted to human topo II α within the perylene-quinones, since the activity of bacterial gyrase was reduced most effectively by the altertoxins ATX I and ATX II, with the same potency. Interestingly, the *Fusarium* mycotoxin DON, also equipped with an epoxy moiety (Fig. 1d), did not show any impact on either human nor bacterial enzyme, possibly due to steric hindrance of the epoxide. Comparison of the two benzo-α-dipyrones, AOH and AME, which are structurally alike except for the additional methyl group in case of AME, showed that this moiety did not affect the inhibitory potency toward topo II (Fig. 1b). In line with these findings, AOH was previously described not only to suppress topo II activity under cell-free conditions but also to act as a topo II poison in intact cells, stabilizing the covalent topo-II-DNA intermediate (Fehr et al. 2009). In contrast, ATX II, also potent under cell-free conditions, did not affect the intracellular levels of the covalent topo-II-DNA intermediate, indicating a function as a catalytic inhibitor (Tiessen et al. 2013).

The impact on the activity of human topo II α, which was observed for 10 out of 15 tested secondary metabolites from *Alternaria* spp. in the decatenation assay, raised the question whether this mechanism was restricted to compounds produced by this genus. As an initial orientating study, considering the amount of known mycotoxins, four *Fusarium* metabolites out of different structural classes (FB1, MON, DON and FC; Fig. 1d) were examined for their effect on human topo II α. Of note, none of the tested *Fusarium* metabolites showed any interference with the enzyme (Fig. 4). Thus, the inhibition of topo II α seemed to be restricted to the secondary metabolites produced by *Alternaria* spp.

The present results indicated a common effect of inhibition of topo II by the tested secondary metabolites produced by *Alternaria* spp. Since topo II is an enzyme family ubiquitous not only in mammals, but plays a vital role in prokaryotes as well, the targeting of topo II by the secondary fungal metabolites may be based on a potential dual inhibition of the human enzyme and its bacterial homologue (Champoux 2001; McClendon and Osheroff 2007). In general, the biosynthesis of substances which interfere with cellular targets of potential predators, that are essential for survival in the food chain, is a mechanism that was described for other fungi imperfecti, as for instance the production of antibiotics by *Penicillium* or *Aspergillus* spp. (Lancini et al. 1995). Indeed, the bacterial topo II, gyrase, is a target of many classes of pharmaceutical antibiotics (Gellert et al. 1976b). In line with this theory, investigations of the impact of the tested secondary metabolites on gyrase from *E. coli* revealed that some of the tested secondary metabolites showed an effect on both the human and bacterial topo II, while other substances were only active against the human enzyme (Table 1). The compounds that suppressed both the human and the bacterial analogue were the perylene-quinone-type mycotoxins and the dibenzo-α-pyrones from *Alternaria* spp. These results support our theory that the impairment of the function of human topo II by secondary fungal metabolites may be attributed to the high degree of homology between human and bacterial enzyme. This implies that gyrase probably constitutes the main target of *Alternaria* spp., but their secondary metabolites affect human topo II as well. Such a dual targeting of topoisomerases was reported for other compounds, for instance the coumarin novobiocin, which targets mammalian as well as prokaryotic topo II (Larsen et al. 2003). Furthermore, several novel quinolone derivatives were shown to be active against both bacterial gyrase and its homolog, also of bacterial origin, the tetrameric topoisomerase IV (Mitton-Fry et al. 2013).

To the best of our knowledge, so far no data about the antibacterial activity of the perylene-quinones or the dibenzo-α-pyrones tested in this study have been published. On the contrary, antibiotic effects were described for some of the metabolites that inhibited human topo II α but not gyrase, to which ALN, ALS, PYR and MAC belong (Fig. 2; Table 1). Antibiotic properties of ALN toward gram-positive bacteria were reported previously, while gram-negative ones, among which *E. coli* are counted, were not affected (Schobert and Schlenk 2008). Furthermore, bacteria possess a second topo II aside from gyrase, topoisomerase IV, which also represents a target of antibiotics (Kato et al. 1990; Khodursky et al. 1995). Although gyrase and topo IV share a high degree of homology, antibacterial agents differ in their sensitivity toward the two enzymes (Hooper 1999; Pommier et al. 2010; Takei et al. 2001). Hence, it is possible to speculate that the compounds that did not affect gyrase, but were active against human topo II, may potentially target topo IV. This specificity for topo IV was also reported for the glycosylated flavonoid quercetin (Bernard et al. 1997). ALS, which inhibited human topo II but not gyrase, might also target topo IV, since the substance was previously reported to decrease the growth of a spectrum of pathogenic bacteria (Kjer et al. 2009).

Only one of the *Alternaria* metabolites, the phytotoxin TEN, and all 4 tested *Fusarium* compounds did not reveal an inhibitory potency against either human or bacterial topo II (Halloin et al. 1970). Interestingly, antibacterial characteristics were described for DON in *Streptococcus agalactaie*, but not *E. coli* (Ali-Vehmas et al. 1998). Presumably the mechanism of action behind the reported antibacterial characteristics was not topo II-related. Generally, the impairment of topo II seemed to be limited to the secondary metabolites from *Alternaria* spp. that were tested in this study, whereas the *Fusarium* mycotoxins did not show any effect on this enzyme, neither on the human nor on the bacterial one.

In conclusion, 10 out of 15 secondary metabolites produced by *Alternaria* spp., evaluated for their impact on human topo II in the present work, revealed an inhibitory potential. Additionally, the perylene-quinone derivatives and the dibenzo-α-pyrones were also active against the bacterial homologue, gyrase, which indicated a dual inhibition of human as well as bacterial topo II. In contrast, none of the tested *Fusarium* metabolites revealed any interference with either human topo II or gyrase. With respect to food safety, interference with topo II apparently has to be considered as a potential genotoxic mechanism for a broader spectrum of *Alternaria* metabolites. On the other hand, in medicinal chemistry, topo II inhibition is a well-used drug effect in cancer therapy. Moreover, targeting of bacterial gyrase is a promising feature toward the identification of novel antibiotic structures. Considering the broad spectrum of secondary metabolites formed by *Alternaria* spp. and the already identified structures with activity toward human topo II and bacterial gyrase, it is likely to speculate that *Alternaria* secondary metabolites might represent a promising pool for the identification of compounds with pharmaceutical potency.

## Electronic supplementary material

Below is the link to the electronic supplementary material.

Supplementary material 1 (PDF 116 kb)
