# Supplementary material for: Dual effectiveness of Alternaria but not Fusarium mycotoxins against human topoisomerase II and bacterial gyrase
Source: Arch Toxicol. 2016 Sep 28;91(4):2007–16. doi: 10.1007/s00204-016-1855-z (PMC5364253; doi:10.1007/s00204-016-1855-z)
Supplement: Supplementary file 1 — Supplementary material 1 (PDF 116 kb) [file 204_2016_1855_MOESM1_ESM.pdf]

Dual effectiveness of *Alternaria* but not *Fusarium* mycotoxins against human topoisomerase II and bacterial gyrase

Katharina Jarolim[1], Giorgia Del Favero[1], Doris Ellmer[2], Timo D. Stark[2], Thomas Hofmann[2], Michael Sulyok[3], Hans-Ulrich Humpf[4], Doris Marko[1*]

[1] University of Vienna, Faculty of Chemistry, Department of Food Chemistry and Toxicology, 1090 Vienna, Austria
[2] Technical University of Munich, Chair of Food Chemistry and Molecular Sensory Science, 85354 Freising, Germany
[3] University of Natural Resources and Life Sciences Vienna (BOKU), Department IFA, Tulln, 3430 Tulln, Austria
[4] Westfälische Wilhelms-Universität Münster, Institute of Food Chemistry, 48149 Münster, Germany

* Corresponding author
Doris Marko
e-mail: doris.marko@univie.ac.at,
tel. nr.: +431427770800

Stemphyltoxin

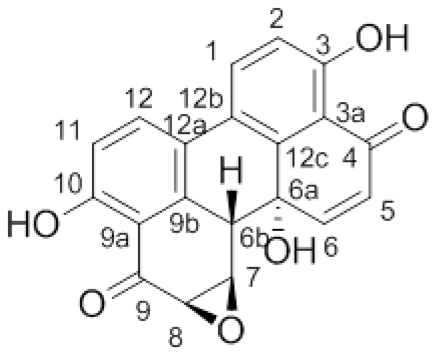

Table S1: 1H NMR data

H	Literature (Me_2CO-d_6), Stack & Mazzola, 1989	NMR-measurement (500 MHz, $CDCl_3$)
1	8.14 d (J=8.8 Hz)	7.92 d (J=8.8 Hz)
2	7.09 d (J=8.8 Hz)	7.13 d (J=8.8 Hz)
3-OH	12.2 s	12.31s
5	6.55 d (J=10.4 Hz)	6.61 d (J=10.4 Hz)
6	7.86 d (J=10.4 Hz)	7.48 d (J=10.4 Hz)
6a-OH	5.15 s	-
6b	3.9 bs	3.81 s
7	4.60 d (J=3.6 Hz)	4.27 d (J=3.2 Hz)
8	3.77 dd (J=3.6, 0.7 Hz)	3.75 d (J=3.0 Hz)
10-OH	12.4 s	12.20 s
11	7.02 dd (J=8.8, 0.8 Hz)	7.07 d (J=8.8 Hz)
12	8.14 d (J=8.8 Hz)	7.92 d (J=8.8 Hz)

Table S2: ^{13}C NMR data

C	Literature (Me_2CO-d_6), Stack & Mazzola, 1989	NMR-measurement (125 MHz, $CDCl_3$)	HMBC coupling with
1	132.7 or 133.7	132.1	3
2	117.4 or 118.8	119.6	3
3	161.8 or 163.6	161.9	1 and 2
3a	113.7 or 115.2	112.6	3, 2 and 5
4	191.0	189.3	6
5	129.3	130.5	-
6	147.8	144.4	-
6a	66.7	66.4	6, 5 and 6b
6b	43.4	43.0	6 and 7
7	57.2	56.2	6b
8	53.5	52.8	6b

9	198.4	196.4	8
9a	113.7 or 115.2	114.9	10, 11, 8 and 6b
9b	140.6	133.7	12, 7 and 6b
10	161.8 or 163.6	163.8	12 and 11
11	117.4 or 118.8	118.2	10
12	132.7 or 133.7	132.8	10
12a	125.0 or 125.8	124.1	1, 11 and 6b
12b	125.0 or 125.8	123.8	2 and 12
12c	136.5	138.1	1, 6 and 6b

Alterperylenol

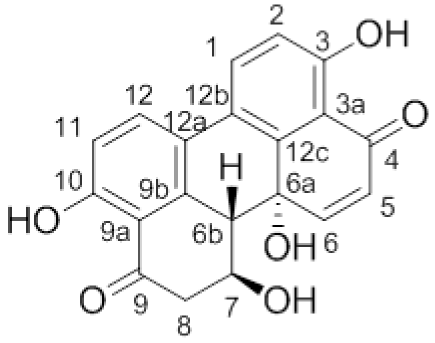

Table S3: ^{1}H NMR data

H	Literature (300 MHz, CD_3CN), Hradil et al. 1989	NMR-measurement (500 MHz, CD_3OD)
1	7.97 d (J=8.8 Hz)	7.95 d (J=8.8 Hz)
2	7.05 d (J=8.8 Hz)	7.04 d (J=8.5 Hz)
3-O	-	-
5	6.32 d (J=10.4 Hz)	6.32 d (J=10.4 Hz)
6	7.84 d (J=10.4 Hz)	7.89 d (J=10.4 Hz)
6a-O	-	-
6b	3.15 dd (J=0.5, 9.8 Hz)	3.15 d (J=9.8 Hz)
7	4.60 ddd (J=5.0, 9.8, 12.0 Hz)	4.60 ddd (J=12.2, 9.7, 5.8 Hz)
7-O	-	-

8-α	2.99 dd (J=5.0, 16.0 Hz)	3.02 dd (J=15.9, 4.9 Hz)
8-β	2.85 dd (J=12.0, 16.0 Hz)	2.87 dd (J=15.8, 12.3 Hz)
10-O	-	-
11	6.96 dd (J=0.5, 8.8 Hz)	6.96 d (J=8.5 Hz)
12	7.92 d (J=8.8 Hz)	8.00 d (J=8.5 Hz)

Table S4: ^{13}C NMR data

C	Literature (25 MHz, $CDCl_3$), Okuno et al., 1983	NMR-measurement (125 MHz, CD_3OD)	HMBC couplings with
1	132.6 or 132.3	132.7	-
2	118.4 or 116.8	118.8	-
3	162.5 or 161.7	162.2	1 and 2
3a	117.6 or 113.5	114.0	2 and 5
4	191.3	191.2	6
5	125.3	126.0	6
6	153.4	153.3	-
6a	67.2	67.6	5 and 7
6b	52.0	52.7	8-a and 8-b
7	65.9	66.3	6b and 8
8	48.0	48.1	7 and 6b
9	204.2	204.6	8
9a	117.6 or 113.5	118.0	11, 6b and 8
9b	140.7 or 138.1	138.2	12and 6b
10	162.5 or 161.7	163.0	11 and 12
11	118.4 or 116.8	117.3	12
12	132.6 or 132.3	133.0	6b
12a	126.4 or 125.3	127.1	11, 1 and 6b
12b	126.4 or 125.3	126.2	12 and 2
12c	140.7 or 138.1	141.2	1, 6 and 6b
